# Patient Characteristics in Persistent Pulmonary Hypertension of the Newborn

**DOI:** 10.1155/2011/858154

**Published:** 2011-05-24

**Authors:** M. T. R. Roofthooft, A. Elema, K. A. Bergman, R. M. F. Berger

**Affiliations:** ^1^Department of Paediatric Cardiology, Beatrix Children's Hospital, University Medical Centre Groningen, University of Groningen, Hanzeplein 1, 9700 RB Groningen, The Netherlands; ^2^Department of Neonatology, Beatrix Children's Hospital, University Medical Centre Groningen, University of Groningen, Hanzeplein 1, 9700 RB Groningen, The Netherlands

## Abstract

*Objective*. To assess the impact of PPHN on mortality, morbidity, and behavioural skills. *Methods*. A retrospective observational study of 143 newborns with PPHN, over an 11-year period, using objective health-status data from medical records and family doctors, and subjective health status data from a standardized Child Behaviour Checklist. *Results*. The majority of patients were males, treated with inhaled nitric oxide had maladaptation/maldevelopment as pathophysiological mechanism and a gestational age >37 weeks. In term newborns, types of pathophysiological mechanism (*P* < .001) and Oxygen Index (*P* = .02) were independent predicting risk factors for PPHN-related mortality. Analysis of preexisting disease and outcome categories in term newborns showed only a significant correlation between the use of iNO and respiratory complaints (*P* = .03), not confirmed by multivariate analysis and regression analysis. *Conclusions*. PPHN is a serious, often fatal condition. The incidence of PPHN in preterm newborns is high. In term survivors, PPHN had no additional role in morbidity/outcome.

## 1. Introduction

PPHN is defined as a failure of normal pulmonary vascular adaptation at or soon after birth, resulting in a persisting high pulmonary vascular resistance such that pulmonary blood flow is diminished and unoxygenated blood is shunted to systemic circulation, via a right-to-left shunting through an open foramen ovale and/or a ductus arteriosus [1]. Potential risk factors, such as prematurity, dysmaturity, infection, meconium aspiration syndrome, genetic anomalies, and structural anomalies, are identified.

PPHN is a complex clinical syndrome with an estimated incidence of 1.9 per 1000 live-births (0.4–6.8/1000 live-births) and a reported mortality rate ranging from 4–33% [2]. Considering the serious nature and mortality rate of PPHN, the interest in the outcome of surviving children is generated. Limited data are available on the long-term outcome of neonates surviving PPHN. Previous studies reported an increased appearance of respiratory tract problems, hearing impairment, delayed mental and motor development and behavioural problems [3–6]. However, these studies were partly hampered by small sample sizes, ranging from 11 to 19 patients, partly dated back to the era before inhaled nitric oxide as treatment modality for PPHN, or limited to term neonates (GA ≥ 37 weeks) [4–7]. 

In this study, we retrospectively describe mortality and morbidity in neonates presenting with PPHN in tertiary cardiac and neonatal intensive care unit. Furthermore, we studied the present health status, including child behaviour scores, of the term surviving neonates in this cohort of PPHN patients.

## 2. Patients and Methods

Between January 1995 and January 2006, 143 patients diagnosed with PPHN, confirmed by echocardiography, were treated consecutively at our institution that serves as a tertiary cardiac and neonatal intensive care unit. All patients were included.

The prenatal, perinatal, and neonatal data of all newborn were collected from the medical record of the patient. Maternal data as age, preexisting diseases, diseases acquired during pregnancy, use of medication, alcohol-, smoking- and drugs-habits, and pregnancy-related complications were obtained by a questionnaire. Patient data, concerning severity and type of pathophysiology of PPHN were determined. Follow-up data of term responding survivors, retrieved from the hospital patient records, as well as the medical records held by the family doctor, contained information about general health status, focussed on respiratory and gastrointestinal tract problems, neurological aspects, such as delayed mental and motor development, hearing- and vision impairment, the use of medication and other supportive treatment, as speech therapy and physiotherapy. 

The parents of the surviving patients (*n* = 92, 64.3%) received the Child Behaviour Checklist. We used a time interval of 6 weeks to respond. The study was approved by the Institutional Medical Ethics Committee and informed consent was obtained from each responding parent.

### 2.1. Child Behaviour Checklist (CBCL)

The CBCL is a standardized measure of child behaviour, developed by Achenbach and normalized for Dutch children by Verhulst et al. Children between 1.5 and 5 years of age received the CBCL for 1.5 to 5 years (CBCL/1.5–5) [8]. Children older than 5 years received the CBCL for 6 to 18 years (CBCL/6–18). The test is designed to assess child's behaviour and social competency, as reported by their parents, implicating a subjective parental opinion. The questionnaire included questions on social issues. Moreover parents are asked to rate their child, on a scale of not true to true on many different issues, including academics, inattention, relationships, and behaviours. In the CBCL/1.5–5 skills are not included.

### 2.2. Definition of PPHN

PPHN was defined based on a combination of clinical and echocardiographical characteristics. Two parameters were obtained to score the severity of PPHN, the preductal to postductal difference in transcutaneous oxygen saturation (*δ*-SO_2_), and the Oxygen Index (OI), respectively. The preductal to postductal difference in transcutaneous oxygen saturation (*δ*-SO_2_) was used to scale severe PPHN (group 1) and mild-moderate PPHN (group 2). Patients with severe PPHN, presented with profound cyanosis, associated with continuous right-to-left shunting, demonstrated by echocardiography (i.e., pulmonary to systemic circulation), through a patent ductus arteriosus (PDA), on colour flow Doppler and pulsed wave Doppler, or a predominant right-to-left shunt through a PDA, associated with a preductal to postductal difference in transcutaneous oxygen saturation (*δ*-SO_2_) of 15% or greater, as measured between a right arm and a leg. In patients with mild-moderate PPHN, bidirectional shunting through the PDA was observed by echocardiography, associated with a *δ*-SO_2_ between 5% and 15% [1]. Secondly, the Oxygen Index (OI) of term newborn children was determined by using the fractional oxygen index (FIO_2_) in percentages times the mean airway pressure in cm H_2_O, divided by the arterial O_2_ pressure in kilopascal, all three values retrieved in the worst stage of the PPHN. An OI of <15 was scored as mild PPHN, between 15–25 as moderate PPHN, between 25–40 as severe, and more than 40 as very severe PPHN [9]. 

### 2.3. Type of Pathophysiological Mechanism

Hypothetically, the pathophysiological mechanisms, responsible for PPHN, are classified into maladaptation, maldevelopment, and underdevelopment, as described in the article of Dakshinamurti [10]. Maladaptation of the normal developed pulmonary vasculature through an imbalance of vasoactive substrates is responsible for the greater part of PPHN and originates often from sepsis, pneumonia, meconium aspiration syndrome, or asphyxia. Abnormal development of the pulmonary vasculature (maldevelopment) is mainly idiopathic but sometimes associated with chronic fetal hypoxia, fetal anaemia, or premature closure of the ductus arteriosus. Lunghypoplasia with underdevelopment of pulmonary vasculature (underdevelopment) originates from several causes, however congenital diaphragmatic hernia or oligohydramnios form the majority of these causes. The discrimination of maladaptation and maldevelopment, however, is difficult or even impossible without histological specimen. Therefore patients were classified into maladaptation/maldevelopment (group 1) and underdevelopment (group 2), based on medical history, clinical course, and additional imaging, as chest X-ray.

### 2.4. Treatment Strategies in PPHN

Therapies for PPHN were aimed at lowering pulmonary vascular resistance and improving mixing at the level of the atria and PDA. The ventilator strategy used, is meant to reduce pulmonary vascular resistance by improving oxygenation while aiming for a pH of 7.4 and an arterial CO_2_ pressure (pCO_2_) between 4.0–5.5 kPa. Ventilator settings were adjusted according to the patient's pulmonary condition, tidal volume, and arterial blood gas determination. Patients received sedation with morphine and, if necessary, neuromuscular blockade with vecuronium. Inotropic agents (isoprenaline, dopamine, dobutamine, and noradrenalin), and intravenous volume replacement were used aggressively to maintain an adequate arterial blood pressure. During iNO therapy, nitric oxide was introduced into the inspiratory limb of the ventilator (5–40 ppm). In case of failure, intravenous vasodilators (tolazoline, epoprostenol, and enoximone) were started in the absence of contraindications (hypotension, renal failure, and haemorrhage) [1]. 

## 3. Statistical Analysis

Data analysis was performed using the SPSS software (version 16; SPSS 2007, Chicago, Illinois, USA). Categorical variables are presented as percentages and numbers. Continuous variables were presented as means (standard deviation) in normally distributed variables. Comparisons between categorical variables were performed using **χ** ^2^ test (independent variables), assessed in mortality and morbidity to sex, severity of PPHN, pathophysiological mechanisms, use of inhaled nitric oxide and gestational age. Depending on the type of variable, a Mann-Whitney *U* test, a Kruskal-Wallis test, a Spearman's rank correlation coefficient, and a *T*-test, one way Analysis of variance were used. In these tests, a *P*-value less than  .05 was considered significant. 

## 4. Results

143 patients with documented PPHN were enrolled in the study. More than one third (36.4%) of the patients had a gestational age (GA) less than <37 weeks (defined as prematurity). In general, the majority of patients were male, treated with inhaled nitric oxide (iNO) and showed maladaptation/maldevelopment as pathophysiological mechanism. In the patient group with a GA < 37 weeks, significantly more children had mild-moderate PPHN, showed underdevelopment as pathophysiological mechanism, and had a higher mortality rate, either related to PPHN or not (Table 1). 

The overall mortality in the patient cohort was high (35.7%), and related to PPHN in more than 31% of the patients.

Six children, 5 boys and one girl, died at a later stage, at a mean age of 3 months (ranging from 10 days to 6 months), independent of PPHN. The reasons of death comprised chronic lung disease, sepsis, cerebral haemorrhage, and ventriculitis. Three children were premature born, two of them suffered from mild-moderate PPHN (Table 2). 

Considering the PPHN-related mortality, we did not observe a difference in gender or severity of PPHN. Inhaled nitric oxide was significantly more used in term newborns (≥37 weeks). A significant majority of pathophysiological underdevelopment was seen in the premature population (<37 weeks), in contrast to the term newborns with significant more maladaptation/maldevelopment (Table 2). Significant correlations were seen between gestational age and the use of iNO, as well as gestational age and type of pathophysiological mechanism, also demonstrated by a multivariate analysis. In a logistic regression analysis Oxygen Index (*P* = .02) and type of pathophysiological mechanism (*P* ≤ .001) are independent predicting risk factors for PPHN-related mortality in term newborns. No other risk-factors for PPHN-related mortality could be identified by multivariate analysis. 

Between premature and term newborns, no difference was observed in comorbidity or presenting symptoms, such as asphyxia, infection, meconium aspiration, or congenital anomalies, except for congenital heart disease (CHD). Four term newborns were diagnosed simple transposition of the great arteries (l-TGA), whereas no premature neonate had a CHD.

## 5. Survivors

The remaining 92 patients (survivors) participated in the cross-sectional observational study, with a mean followup of 5.6 years (SD = 3 yrs), ranging from 0 to 11 years. Two patients were untraceable. Child Behaviour Checklists (CBCLs) were sent to the 90 survivors. The response rate was 61.1%  (*n* = 55). Sixteen patients had a gestational age less than 37 weeks, 39 patients more than 37 weeks. The mean age in responders (4.89 years, SD = 2.62) is significantly lower than that in nonresponders (6.65 years, SD = 3.19) (*P* < .01). The severity of PPHN and type of pathophysiology showed no significant differences between responders and nonresponders. All 55 responding families had the Dutch nationality. Thirty-seven responding families represent male patients (37/55, 67%). Only the data of the responders were used for analysis. In 51 of the 55 patients, hospital records and family doctor data (FD data) were available. Basic characteristics of all surviving patients are shown in Table 3.

## 6. Morbidity

The relation of PPHN to morbidity could be blurred by coexisting diseases, sometimes directly related to therapy, as in mechanical ventilation or maturation disorders in premature newborns. Therefore, only the data of term neonates, without syndromal anomalies, were evaluated, in order to expel these coexisting symptoms. Basic characteristics are shown in Table 4. 

In the term newborns, more than one-third of the patients frequently use medication. Respiratory tract problems, like asthma and pneumonia, is an important contributor of morbidity, for which among others bronchodilators (*n* = 10) and inhaled steroids (*n* = 5) are used (Table 5). Hearing impairment, mentioned by the parents, was seen in five children (14.3%), somewhere in followup, with permanent loss of high-frequency sound in two children and combined permanent loss of high- and low-frequency sound in one child. Nevertheless no patient is using a hearing aid. Moreover, the medical records listed no hearing impairment. Hearing impairment is seen in 3% of regular school going children (age 4–18 years) in The Netherlands. Five children (14.3%) have a visual impairment, that is, far-sightedness (*n* = 2), myopia (*n* = 2), and strabismus (*n* = 1). Four children have glasses/contact lenses. Visual impairment is observed in 2–6% of the overall Dutch paediatric population. 

For identifying the pathological role of PPHN on outcome, an artificial division in preexisting disease (comorbidity categories: asphyxia, infection, MAS, CHD and the combination of MAS, and infection) and morbidity (outcome categories: respiration, CHD, major neurology and visual and hearing impairment) was created. In a univariate analysis of comorbidity categories and outcome categories, we observed only a significant correlation between the use of iNO and respiratory complaints (Pearson correlation = 0.65, *P* = .03), suggesting a causal role for PPHN in outcome of term patients. However, this causal role was not confirmed in a multivariate analysis and regression analysis of preexisting disease and outcome (*F* = 5.8, *P* = .003).

## 7. Child Behaviour Skills

In the analysis of child behaviour and social competency, the results of the CBCL of PPHN survivors (*n* = 55), reported by their parents, were used. In general, parents asses their children as having an optimal behaviour in 85.7% (CBCL/1.5–5, 24/28 responders) and 81% (CBCL/6–18, 17/21 responders), whereas in 14.3% (CBCL/1.5–5, 4/28 responders) and 19.1% (CBCL/6–18, 4/21 responders) the child behaviour was scored as abnormal or at risk. Remarkable is the large portion of abnormal social skills (57.9%, 11/19 responders) in children between 6 and 18 years. These skills are not investigated in the CBCL/1.5–5 [8]. 

## 8. Maternal Data

The overall mean age of mothers (*n* = 143) at delivery is 31 years (SD = 4 yrs), ranging from 21 to 43 years and did not differ from the average age of pregnant women in The Netherlands of 30.7 years (2000) and 31.1 years (2006), according to the Dutch Central Registration Agency (CBS). 

Hospital records of the 55 responding mothers, demonstrated six mothers with preexisting morbidity, such as systemic hypertension, migraine, and diabetes. 15 mothers experienced diseases during pregnancy, especially systemic hypertension (*n* = 11), pre-eclampsia (*n* = 4), and gestational diabetes mellitus (*n* = 2). Complications were listed in 22 pregnancies, such as premature rupture of membrane (*n* = 5), premature contractions (*n* = 3), intrauterine growth retardation (*n* = 2) and vaginal blood loss (*n* = 4) with placental solution in two mothers. 

Nineteen mothers used medication during pregnancy, like antihypertensive agents (*n* = 4), insulin (*n* = 4), antibiotics (*n* = 2), and iron replacement therapy (*n* = 3). In contrast to earlier publications, no mother was using a serotonin-reuptake inhibitor [11, 12]. 

## 9. Discussion

In this study we retrospectively investigated characteristics of neonates suffering from PPHN, in tertiary cardiac and neonatal intensive care unit. Subsequently, we studied the health status of the survivors in this cohort, over an 11-year period.

In our study cohort, more than one third of the patients were premature newborns (GA < 37 weeks), taking into account the incidence of prematurity in the general Dutch population (7.8%, Perinatal Care 2003). The majority of patients were boys, were treated with iNO, and had maladaptation/maldevelopment as pathophysiological mechanism. In the group of premature newborns with significantly more children with mild-moderate PPHN, we observed a higher mortality rate, either related to PPHN or not, compared to term newborns. Moreover, underdevelopment as pathophysiological mechanism was seen significantly more often in premature babies. The overall mortality was high (Table 1), though corresponding to the literature (11%, range 4–33%). 

Focussing on term newborns, type of pathophysiological mechanism (*P* < .001) and Oxygen Index (*P* = .02) were the only independent predicting risk factors for PPHN-related mortality (regression analysis). We should make a comment concerning the Oxygen Index in our term newborn population, containing 14 children with a transposition of the great arteries. In this congenital heart defect, the Oxygen Index, does not represent a parameter for respiratory function, but more a parameter for mixing of the blood pool and the circulation. Taking this into account, the Oxygen Index could be of more importance for the remaining term study population. 

In the literature, concerning outcome and health-status of PPHN patients, we can distinguish two episodes, namely, the publications originating from the preinhaled nitric oxide era (pre-NO-era), and the literature from the inhaled nitric oxide era (NO-era) [3–7, 13]. In the pre-NO-era Ballard reviewed the current literature at that time, concerning the effect of hyperventilation in PPHN patients, focussed on mental and psychomotor development. Thirty-six patients, coming from three studies (Breet (1981), Bernbaum (1981) and Ferrara (1983)), showed, in a followup ranging from 12 to 35 months, normal neurological outcome in 32 patients, scored by Mental Development Index (MDI) and Psychomotor Development Index (PDI), both as part of the Bayley assessment (first edition). In their own cohort Ballard studied 11 patients, all term babies, 9 patients were evaluated at the age of 2 years, with normal neurological outcome in 8 patients [13]. One child had some delays in fine motor development. Leavitt and coworkers looked, in the pre-NO era, at 12 patients, all term- or near-term neonates with PPHN, who were on mechanical ventilation at least 72 hours, with a mean age at followup of 20 months, ranging from 12 to 26 months. Neurodevelopment examination, Bayley assessment included, and visually reinforced audiometry (VRA) were performed. Four children showed abnormal neurodevelopment, including three children with sensorineurinal hearing impairment [5]. From the NO era, we considered three studies by Ichiba et al., Lipkin et al., and Ellington et al. as relevant in the comparison to our study [3, 4, 6]. Ichiba et al. investigated 18 term and near-term neonates with PPHN treated with iNO (≥34 weeks gestational age). They divided their patients into three groups on the basis of response to iNO, namely, early response in 8 patients, late response in 8 patients, and poor response in 8 patients. The mortality rate was 16.7%; all patients belonged to the poor response group. The initial dose of iNO was 10 parts per million (ppm), if needed increased to 40 ppm. Followup, at three years, showed reactive airway disease in 5 patients, no loss of sensorineurinal hearing and a significant higher incidence of normal neurodevelopment outcome in the early response group, however only one patient was identified as having a mild neurodevelopment disability [4]. In the study of Lipkin 155 term neonates (GA ≥ 37 weeks) were checked, at the age of one year. Patients were divided in two groups (iNO versus placebo). In 144 patients surviving patients, the second Bayley Scales of Infant Development (second edition), neurological examination, sound field audiometry, and tympanometry were performed. No significant differences between the placebo and iNO groups were seen in any long-term outcome. There were major neurological abnormalities in 13%, cognitive delays in 30%, and hearing loss in 19% of the infants. Apart from the short follow-up period, patients with lunghypoplasia syndromes, chromosomal abnormalities, intracranial haemorrhage (≥grade 2), or patients treated with surfactant or high frequency oscillation ventilation were excluded [6]. Finally Ellington et al. looked at 60 patients, surviving PPHN, with a follow-up interval ranging from 1 to 4 years. The gestational age ranged from 34 to 43 weeks. Parents were interviewed by telephone, using a trained interviewer and standardized instruments. The overall neurological handicap rate was 15%, a hearing deficit in 7%, and behavioural problems in 26%. No adverse health or neurodevelopment outcomes have been observed among infants treated with nitric oxide for PPHN, as later reported by Ellington et al. [3] and Konduri and coworkers [14].

The relation of PPHN to morbidity could be blurred by coexisting diseases. Therefore, only the data of term neonates, without syndromal anomalies, were evaluated, trying to expel these coexisting symptoms. In our term responding newborns with PPHN, respiratory problems (34.3%; 12/35) were frequently seen, in comparison to the prevalence of asthma, chronic bronchitis, and emphysema in 10% of the Dutch children between 4 and 12 years of age (according to the CBS, Dutch Central Registration Agency 2008). Seven preterm children suffered from chronic lung disease (43.8%; 7/16), for which a daily need of bronchodilators existed. A high prevalence of visual impairment of 14.3% (5/35) was observed, in contrast to the 2–6% in the general Dutch children population. Though, a significant relation between visual impairment and PPHN could not be demonstrated in our cohort. Hearing impairment in our study comprises 14.3% (5/35) of the children, obtained by the questionnaires, remarkably not confirmed by the medical records or family doctor data. In two of the 5 patients, the hearing impairment is permanent, although no patient is using a hearing aid. The incidence of hearing impairment, observed by the Dutch school doctors, in children between 4 and 18 years is 3%. The majority of these hearing complaints are temporarily, often due to ear infections. The literature mentions various figures of incidence, ranging from 0 to 52.5% [3, 4, 6, 15–17]. 

The prevalence of impaired motor development, caused by delayed motor development (40%) and cerebral palsy (25.7%) is comparable to the results in early reports, concerning PPHN (15–33%), however being high in comparison to the general Dutch children population (<1%). Furthermore, Boys showed significant more delayed neuromotor development than girls, corresponding with results in earlier reports.

In a univariate analysis of comorbidity categories and outcome categories, we observed only a significant correlation between the use of iNO and respiratory complaints (Pearson correlation = 0.65, *P* = .03), suggesting a causal role for PPHN in outcome of term patients. However, this causal role was not confirmed in a multivariate analysis and regression analysis of preexisting disease and outcome (*F* = 5.8, *P* = .003), suggesting no additional causal role of PPHN in outcome of term newborns.

Using the results of the CBCL, we first should acknowledge its highly subjective nature. However, we registered an incidence of 4.1% (2/49) of abnormal behaviour and 12.2% (6/49) of the children show behaviour at risk. These results correspond with the general prevalence of 5–15% of Dutch children with mild-to-moderate behavioural problems. In contrast, Ellington and coworkers observed significant behavioural problems in 26% of the children, surviving PPHN. In their study, the follow-up interval was limited, ranging from 1 to 4 years, whereas the gestational age ranged from 34 to 43 weeks. Parents were interviewed by telephone, using a trained interviewer and standardized instruments [3]. The CBCL provides information concerning socioemotional performance/skills of children, scored in the complete paediatric population, only possible to test in older children (CBCL/6–18). We were able to obtain these scores in 19 patients, which showed a distinct high proportion of abnormal skills in 57.9%  (*n* = 11).

Considering our methodology, we were faced with limitations. The lack of a case-matched study population forces us to compare patients and their morbidity and behavioural skills with children out of the general dutch population, measured by national health registries, such as PGO, a Dutch program of Preventive Healthcare Statistics, CBS, the Dutch Central Registration Agency and Prenatal Care, a register for neonatal health aspects. Secondly, the use of questionnaires could lead to a response bias. We noticed a significant difference in age of responders, as well as the fact that all responders having the Dutch nationality. Parental remembrance and response could be higher in younger patients and in cases with a more severe clinical course, although, in retrospect, no significant differences in severity and type of pathophysiological mechanism were observed between responders and nonresponders.

## 10. Conclusion

In this combined retrospective and prospective cross-sectional observational study 143 consecutive newborns with PPHN were studied over an 11-year period. In contrast to early publications, we experienced a large contribution of premature neonates. In our series, PPHN was a serious and often fatal condition, associated with a high mortality (31.5%) and morbidity (64.7%). Above all the application of inhaled nitric oxide did not improve the mortality rate or morbidity. Mortality was correlated with the pathophysiology (*P* < .01) and severity (*P* = .05) of PPHN. Morbidity figures in survivors (response rate 61.1%) revealed a long-term morbidity during followup, represented by respiratory problems, delayed/impaired motor development, cerebral palsy, and vision impairment. The use of medication is high, as well as the appealing for health care. However, in term newborns, the pathological role of PPHN on morbidity, could not be proven. Outcome and morbidity was directly related to the preexisting disease. 

Further study, preferably a matched-control prospective study with physical examination and observation, is needed to explore different aspects of morbidity in relation to PPHN more thoroughly.

## Figures and Tables

**Table 1:** Baseline characteristics of PPHN-patients.

*N* (%)	All newborns (*N* = 143)	GA ≥ 37 weeks (*N* = 91)	*GA* < 37 weeks (*N* = 52)
Gender			
Male	86 (60,1)	52 (57,1)	34 (65,4)
Use of iNO	95 (67,4)	65 (73)	30 (57,7)
Pathophysiology			
Maladap/maldevelop	108 (75,5)	77 (84,6)	31 (59,6)*
Underdevelopment	35 (24,5)	14 (15,4)	21 (40,4)*
Severity PPHN			
*δ*-SO_2_			
Mild-moderate	81 (56,6)	43 (47,3)	38 (73,1)*
Severe	62 (43,4)	48 (52,7)	14 (26,9)*
Outcome			
Overall mortality	51 (35.7)	26 (28.6)	25 (48.1)*
PPHN-related mortality	45 (31.5)	23 (25.3)	22 (42.3)*

GA = gestational age, iNO = inhaled nitric oxide, maladap = maladaptation, maldevelop = maldevelopment, statistic significance (*P* < .05, *Chi square test).

**Table:** (a)

PPHN-related mortality (*n*)	All newborn	<37 weeks	≥37 weeks	*P*-value
Total	45	22	23	
Female	22	11	11	
Inhaled NO	31	11	20	.03
Severity PPHN (*δ*-SO_2_)				
Severe/severe + iNO	32/25	16/9	16/14	
Mild-moderate/mild-mod + iNO	13/8	6/2	7/6	
Severity (OI)				
Mild <15			2	
Moderate 15–25			2	
Severe 25–40			5	
Very severe >40			14	<.001
Pathophysiology				
Maldevelop/maladap	20	5	15	.004
Maldevelop/maladap + iNO	15	2	13	.001
Underdevelopment	25	17	8	.004
Underdevelopment + iNO	16	9	7	

**Table:** (b)

Non-PPHN-related morality					
Patient	Severity PPHN	Pathophysiology	Gestational age	Cause of death	iNO

(1) ♂	Severe	Maladap/maldev	39^+3^ wks	Chronic lung disease	−
(2) ♀	Severe	Maladap/maldev	29^+3^ wks	Chronic lung disease	**−**
(3) ♂	Severe	Underdevelopment	40 wks	Sepsis	**+**
(4) ♂	Mild-moderate	Maladap/maldev	38^+6^ wks	Cerebral haemorrhage	**+**
(5) ♂	Mild-moderate	Maladap/maldev	29^+6^ wks	Ventriculitis	**−**
(6) ♂	Mild-moderate	Underdevelopment	28^+5^ wks	Chronic lung disease	**−**

Maladap/maldev = maladaptation/maldevelopment, iNO = inhaled nitric oxide.

**Table 3:** Patient characteristics of surviving patients.

Gestational Age (GA)	<37 weeks	≥37 weeks	*P*-value
Total	27	65	
Female	6	29	.04
Inhaled NO	18	44	
Severity PPHN			
Severe	17	31	
Severe (iNO)	11	21	
Mild-moderate	10	34	
Mild-moderate (iNO)	7	23	
Severity (OI)			
Mild <15		13	
Moderate 15–25		17	
Severe 25–40		18	
Very severe >40		14*	
Pathophysiology			
Maldevelop/maladap	22	60	
Maldevelop/maladap + iNO	15	42	
Underdevelopment	5	5	
Underdevelopment + iNO	3	2	
Comorbidity (*n*/%)			
CHD (TGA)	1	14	.03
Asphyxia	2	10	
Lunghypoplasia nos	4	0	.001
Lunghypoplasia (neuromuscular)	0	0	
Lunghypoplasia (omphalocele)	0	0	
Lunghypoplasia/CDH	0	5	
Lunghypoplasia/cystic kidneys	1	0	
Infection	5	11	
Emphysema	1	0	
Hydrops fetalis (unknown origin)	0	0	

OI = oxygen index, Maldevelop/maladap = maladevelopment/maladptation, CHD = congenital heart disease.

**Table 4:** Patient characteristics of term surviving patients.

Surviving term responders	*n* (%)
Total	35 (100%)
Female	18 (51.4%)
iNO	25 (71.4%)
Maladaptation/maldevelopment	35 (100%)
Severity PPHN	
*δ*-SO_2_	
Mild-moderate	18 (51.4%)
Severe	17 (48.6%)
Severity OI	
Mild <15	5 (14.3%)
Moderate 15–25	10 (28.6%)
Severe 25–40	11 (31.4%)
Very severe >40	9 (25.7%)
ECMO	3 (8.6%)
HFOv	5 (14.3%)
Comorbidity	
Asphyxia	8 (22.9%)
Infection	8 (22.9%)
MAS	10 (28.6%)
CHD	7 (20.4%)
Combination MAS/Asphyxia	2 (5.7%)

iNO = inhaled nitric oxide, OI = oxygen index, ECMO = extracorporeal membrane oxygenation, MAS = meconium aspiration syndrome, CHD = congenital heart disease, HFOv = high frequency oscillation ventilation.

**Table 5:** Morbidity status of term survivors.

Morbidity	Medical data (*n* = 35)	Questionnaires (*n* = 35)	*P*-value
Medication use	12 (34.3%)	9 (25.7%)	
Respiratory tract problems	10 (28.6%)	10 (28.6%)	
Gastrointestinal tract problems	2 (5.8%)	3 (8.6%)	
Congenital heart disease	7 (20%)	7 (20%)	
Speech therapy	8 (22.9%)	12 (34.3%)	
Physiotherapy	9 (25.7%)	14 (40%)	
Delayed socioemotional development	3 (8.6%)	0	
Delayed mental development	7 (20%)	2 (5.8%)	
Microcephaly	1 (2.9%)	1 (2.9%)	
Seizures	1 (2.9%)	1 (2.9%)	
Cerebral palsy	9 (25.7%)	9 (25.7%)	
Tetraplegia	1 (2.9%)	1 (2.9%)	
Hypotonia	2 (5.8%)	2 (5.8%)	
Hearing impairment	0	5 (14.3%)	.02
Visual impairment	5 (14.3%)	6 (17.1%)	
Delayed motor development	14 (40%)	3 (8.6%)	.002
